# Application of a Radiomics Machine Learning Model for Differentiating Aldosterone-Producing Adenoma from Non-Functioning Adrenal Adenoma

**DOI:** 10.3390/bioengineering10121423

**Published:** 2023-12-14

**Authors:** Wenhua Yang, Yonghong Hao, Ketao Mu, Jianjun Li, Zihui Tao, Delin Ma, Anhui Xu

**Affiliations:** 1 Department of Radiology, Tongji Hospital, Tongji Medical College, Huazhong University of Science and Technology, Wuhan 430030, China; ywh1997666@163.com (W.Y.); hyh.0000@163.com (Y.H.); muketao@163.com (K.M.); lijianjun@hust.edu.cn (J.L.); TZH19960520@163.com (Z.T.); 2 Department of Endocrinology, Tongji Hospital, Tongji Medical College, Huazhong University of Science and Technology, Wuhan 430030, China

**Keywords:** radiomics, machine learning, adrenal incidentaloma, aldosterone-producing adenoma, non-functioning adrenal adenoma

## Abstract

To evaluate the secretory function of adrenal incidentaloma, this study explored the usefulness of a contrast-enhanced computed tomography (CECT)-based radiomics model for distinguishing aldosterone-producing adenoma (APA) from non-functioning adrenal adenoma (NAA). Overall, 68 APA and 60 NAA patients were randomly assigned (8:2 ratio) to either a training or a test cohort. In the training cohort, univariate and least absolute shrinkage and selection operator regression analyses were conducted to select the significant features. A logistic regression machine learning (ML) model was then constructed based on the radiomics score and clinical features. Model effectiveness was evaluated according to the receiver operating characteristic, accuracy, sensitivity, specificity, F1 score, calibration plots, and decision curve analysis. In the test cohort, the area under the curve (AUC) of the Radscore model was 0.869 [95% confidence interval (CI), 0.734–1.000], and the accuracy, sensitivity, specificity, and F1 score were 0.731, 1.000, 0.583, and 0.900, respectively. The Clinic–Radscore model had an AUC of 0.994 [95% CI, 0.978–1.000], and the accuracy, sensitivity, specificity, and F1 score values were 0.962, 0.929, 1.000, and 0.931, respectively. In conclusion, the CECT-based radiomics and clinical radiomics ML model exhibited good diagnostic efficacy in differentiating APAs from NAAs; this non-invasive, cost-effective, and efficient method is important for the management of adrenal incidentaloma.

## 1. Introduction

Adrenal incidentaloma (AI) is a type of tumor affecting the adrenal gland and is often discovered in physical examinations or during surgical treatments for a non-adrenal disease [1,2]. The increasing reliance on computed tomography (CT), magnetic resonance imaging, and positron emission tomography-CT, as well as the increasing frequency of physical examinations, has led to higher AI detection rates [3]. Primary aldosteronism (PA) is mainly caused by aldosterone-producing adenomas (APAs) and adrenal hyperplasia. APAs mostly occur unilaterally and are usually surgically removed to achieve a clinical cure [4,5]. PA is the most common cause of secondary hypertension and can occur in 5–10% of cases of essential hypertension (EH) [6]. In China, approximately 44.7% of adults have some form of hypertension. Based on the 5–10% prevalence of PA, there are approximately 12 million patients with the disease, including those with potentially curable subtypes [5]. In addition to hypertension, PA also can lead to harmful metabolic and pathophysiology alterations, especially in patients with cardiovascular and kidney disease [7].

Upon initial AI diagnosis during a physical examination in a patient with hypertension, it can be uncertain whether the tumor is a functional adenoma, and further clinical screening is needed to determine the secretory function of the tumor. For patients with a positive screening result, adrenal venous sampling (AVS) or other techniques are performed to facilitate a definitive diagnosis of the disease subtype [8]. AVS is an invasive procedure that requires the technical competence of interventional radiologists; therefore, AVS is only performed in large hospitals, and the patient may experience adverse effects associated with the additional radiation exposure [9,10]. Therefore, some studies have explored the use of non-invasive and convenient examination methods to mitigate such risks in this patient population.

The accurate prediction of the AI pathology before surgery is crucial to achieve predictive, preventive, and personalized treatment [11]. An increasing number of studies have recently investigated the application of radiomics-based approaches to diagnose afflictions of the adrenal glands. Radiomics has the ability to predict adrenocortical carcinomas, metastatic carcinomas, pheochromocytomas, and APAs by quantitatively extracting the features of adrenal lesions before surgery [12,13,14,15]. One study reported that contrast-enhanced CT (CECT)-based radiomics can be used to identify non-functioning adrenal adenomas (NAAs) in patients with EH and APAs in patients with PA [16]. However, the study only included patients with EH with NAA because some subclinical PA patients have APA tumors, but the clinical manifestations are normal; that is, there are no or only mild symptoms of hypertension and hypokalemia [17]. In our study, we included not only patients with abnormal baseline blood pressure, but also patients with normal baseline blood pressure. Therefore, the purpose of the present study was to investigate whether a logistic regression machine learning (ML) model based on radiomics combined with baseline clinical characteristics could distinguish APAs from NAAs to, ultimately, develop a non-invasive, cost-effective, and efficient technique for the preliminary determination of the secretory function of AIs. For the convenience of clinicians, we also generated an online prediction model. Further, such a model could facilitate the further clinical examination and follow-up treatment of patients with AI.

In this article, we first described the inclusion of cases, and provided a grouping and statistical analysis of cases. Then, the image acquisition, radiomics features selection, and logistic regression ML model construction were described. Finally, the performance of the model was evaluated, and the interpretability of the model was described.

## 2. Materials and Methods

### 2.1. Study Design and Patients

This retrospective study was approved by the Institutional Review Board of Tongji Hospital of Tongji Medical College of Huazhong University of Science and Technology Board (TJ-IRB20221103). The requirement for informed consent was waived because of the retrospective nature of the study. The study conformed to the tenets of the Declaration of Helsinki.

**Figure 1:**
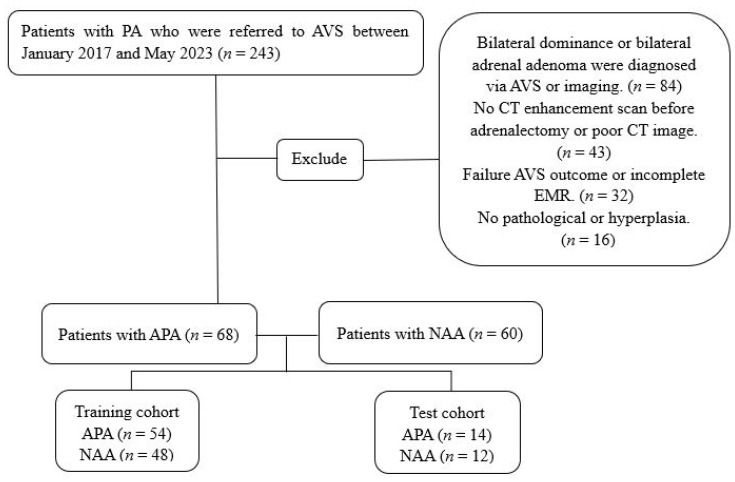
Flowchart of the patient inclusion process.

Overall, 243 patients with PA who underwent AVS at our hospital between January 2017 and May 2023 were retrospectively evaluated. The inclusion criteria were a biochemical diagnosis of PA according to the Endocrine Society guidelines and undergoing AVS prior to surgery [8,18]. The exclusion criteria were as follows: (1) bilateral dominance or bilateral adrenal adenoma diagnosed by using AVS or imaging (*n* = 84); (2) failure to perform CECT imaging before adrenalectomy or poor CT image quality (*n* = 43); (3) failed AVS procedure or incomplete electronic medical records (*n* = 32); and (4) no evidence of pathology or pathological hyperplasia (*n* = 16). A total of 68 patients were included in the study after exclusions. Sixty patients with unilateral NAA who were treated at our hospital comprised the control group. The patients were randomly assigned to the training and test cohorts in an 8:2 ratio. The patient selection flowchart is shown in Figure 1.

APA was diagnosed according to the following criteria: (1) evidence of nodule or adenoma on preoperative CECT; (2) presence of the adenoma on the dominant side, as confirmed via AVS (basic lateralization index > 2 or >4 after adrenocorticotropic hormone stimulation); (3) confirmation of adrenocortical adenoma based on pathological examination; and (4) improvement of blood pressure following surgery [18,19]. NAA was diagnosed according to the following parameters: (1) imaging diagnosis of unilateral adrenal adenoma with benign lesion features and (2) all biochemical indices assessed during clinical screening being within the normal parameters [20].

### 2.2. Image Acquisition

CECT was performed in all patients before surgery (CT scanners used: Toshiba Aquilion One 320 CT, Toshiba, Tokyo, Japan; GE Optimal CT 680/660 Series, GE Healthcare, London/UK; ICT256, Philips, Amsterdam, The Netherlands; SOMATOM Force, Siemens, Berlin, and Munich/Germany). The scanning parameters were as follows: tube voltage of 100–120 kV; slice thickness of 0.625 mm to 1.5 mm; and tube current equipped with automatic mAs technology. Ultravist contrast medium (Bayer Schering Pharma AG, Berlin, Germany) was administered intravenously (rate: 5–6.5 mL/s; concentration: 370 mg/mL). The amount of contrast medium to be administered was calculated according to each patient’s weight and height. Subsequently, 30 mL of saline was administered for flushing under the same injection conditions used for the contrast medium, and images of the adrenal gland were captured following a delay of 40 s.

### 2.3. Extraction of Radiomics Features

**Figure 2:**
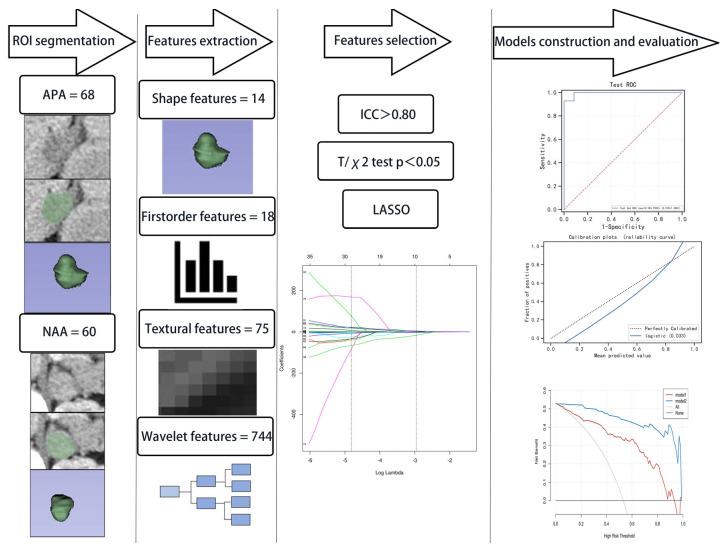
The workflow of this research.

The region of interest covering the entire adrenal adenoma on each consecutive slice was segmented by a radiologist through density threshold-assisted manual segmentation using a 3D Slicer (version 4.11.20210226, RRID:SCR_005619). During the delineation process, vascular shadows, obvious necrotic cysts, surrounding adipose tissue, and other organ tissues were avoided. Three-dimensional images were reconstructed, and radiomics features were extracted using the “Radiomics” extension of the 3D Slicer The images were resampled to 1 × 1 × 1 mm^3^ voxels using linear interpolation. The levels and windows of all CECT images were standardized to 40 and 350 Hounsfield units (Hu), respectively. A cohort of 20 patients was randomly selected from those with APA to evaluate the test–retest reliability of the extracted features after secondary segmentation by the same reader. The time elapsed between the first and second rounds of segmentation was approximately 1–2 months. The workflow of this research is illustrated in Figure 2.

### 2.4. Selection of Radiomics Features and Model Development

The significant radiomics and clinical features in the training cohort were selected according to the following steps. First, the intra-class correlation coefficient (ICC) was calculated, and the features with ICC > 0.80 were retained and compared using either the Student’s *t* test or χ^2^ test. To further reduce the number of redundant features, a third round of feature screening was conducted using the least absolute shrinkage and selection operator (LASSO) regression. Finally, the optimal radiomics features were screened, and a radiomics score tag (Radscore) was calculated according to Formula (1) to build the Radscore ML logistic regression model. After the Student’s *t* or χ^2^ tests were performed for the basic clinical characteristics, LASSO regression analysis was performed for baseline clinical features and the Radscore. The significant variables were used to construct the Clinic–Radscore ML logistic regression model, which was based on a combination of clinical features and Radscore.

Radscore = β0 + β1 × X1 + β2 × X2 +…+ βm × Xm.(1)

### 2.5. Model Evaluation and Interpretation

The classification performances of the Radscore and Clinic–Radscore models were assessed through the receiver operating characteristic (ROC) curves, calibration curves, and decision curve analysis (DCA). The DeLong test was conducted to compare the significance of the area under the curve (AUC) of the ROC curves between the Radscore and Clinic–Radscore models. The calibration curves were used to assess the extent to which the model predictions deviated from actual events. DCA was conducted to evaluate the clinic utility of the Radscore ML model and Clinic–Radscore ML model by quantifying the net clinical benefits at different threshold probabilities, as previously described [21]. In addition, the accuracy, sensitivity, specificity, and F1 score were assessed for the Radscore and Clinic–Radscore ML model in the training and validation and test cohorts. The Shapley Additive Explanation (SHAP), a reliable, fast, and convenient method, was used to explain the output of ML [22]. Each predictor was ranked in order of importance based on the SHAP values.

### 2.6. Statistical Analysis

For the missing baseline clinical data, this study adopted a median interpolation, and the total number of all missing data was <2%. We used ML logistic regression to develop the models. A 5-fold cross-validation was used for the inner validation of the models. The packages used are shown in Table 1. The clinical baseline characteristics were analyzed in the training set and test set and in APA patients and NAA patients in the training set. All statistical analyses were performed using the IBM SPSS Statistics (version 25.0, RRID:SCR_016479) and R Project for Statistical Computing (version 3.6.8, RRID:SCR_001905), and Python (version 3.7, Python Software Foundation, New York, NY, USA). A two-sided *p* value of <0.05 was considered statistically significant.

## 3. Results

### 3.1. Patient Characteristics

A total of 68 patients with APA and 60 patients with NAA were included in this study. The APA group included 29 males and 39 females, and the age ranged from 16 years to 69 years. The NAA group included 31 males and 29 females, and the age ranged from 28 years to 67 years. Overall, 102 patients comprised the training cohort (median age: 51 years; range: 24–69 years; 50 males and 52 females), and 26 patients comprised the test cohort (median age: 48.5 years; range: 16–66 years; 10 males and 16 females).

The baseline clinical characteristics of all patients are shown in Table 2. There were no significant differences in the clinical variables between the training and test cohorts (*p* ≥ 0.05), confirming equal random allocation. The comparison of the baseline patient characteristics between the APA and NAA groups in the training cohort is shown in Table 3. Age, duration of time since the discovery of hypertension (Time), systolic blood pressure (SBP), diastolic blood pressure (DBP), potassium level, and Radscore were significantly different between the groups (*p* < 0.001). However, sex, fasting plasma glucose, total cholesterol, serum creatinine levels, estimated glomerular filtration rate, and body mass index were not (*p* ≥ 0.05).

### 3.2. Selection of Radiomic Features and Model Development

**Figure 3:**
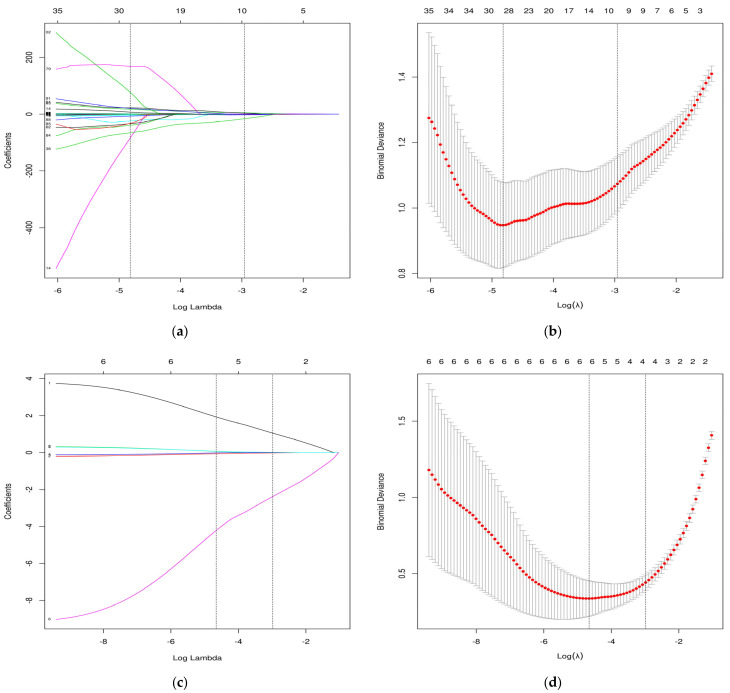
Selection of the aldosterone-producing adenoma candidate variables by LASSO regression. (**a**) LASSO coefficient profiles of the ten candidate factors of radiomics. (**b**) The optimal tuning parameter (*λ*) in the LASSO model of radiomics. (**c**) LASSO coefficient profiles of the four candidate factors of the Clinic–Radscore model. (**d**) The optimal tuning parameter (*λ*) in the LASSO model of the Clinic–Radscore model.

In total, 128 maps (68 APAs and 60 NAAs) were used, and 851 radiomics features were extracted per map using the 3D Slicer. In the first step, 389 unstable features were screened out based on the ICC results, and 462 robust features were retained. In the second step, 268 non-significant radiomics features were screened out based on the Student’s *t*-test or χ^2^ test results (*p* > 0.05), and 194 radiomics features were retained. In the third step, the 194 radiomics features were further reduced via LASSO regression analysis, and the ten optimal radiomics features were used to construct the Radscore model according to Formula (2). The dimensionality reduction process based on the LASSO regression was illustrated in Figure 3a,b. The LASSO regression analyses of the Radscore, age, Time, SBP, DBP, and potassium levels are shown in Figure 3c,d. The Radscore, age, DBP, and potassium level were finally retained and used to construct the Clinic–Radscore ML logistic regression model. The parameters of the logistic regression model were set to regularization factor (C = 1.0), number of iterations (max iter = 100), regularization type (penalty = I2), and convergence measure (tol = 0.0001).

Radscore = 9.366 − 0.319 × (original_(shape_Flatness)) +0.739 × (original_(shape_SurfaceVolumeRatio))
+0.007 × (wavelet-LLH_(firstorder_10Percentile))
+0.007 × (wavelet-LLH_(gldm_LargeDependenceLowGrayLevelEmphasis))
−0.557 × (wavelet-LLH_(glszm_ZoneEntropy)) + 4.712 × (wavelet-LHL_(glcm_Imc2))
−0.044 × (wavelet-LHL_(ngtdm_Busyness)) − 2.578 × (wavelet-LHH_(glrlm_RunEntropy))
−16.345 × (wavelet-HHL_(gldm_DependenceNonUniformityNormalized))
+3.628 × (wavelet-LLL_(glcm_MaximumProbability))(2)

### 3.3. Model Evaluation and Interpretation

**Figure 4:**
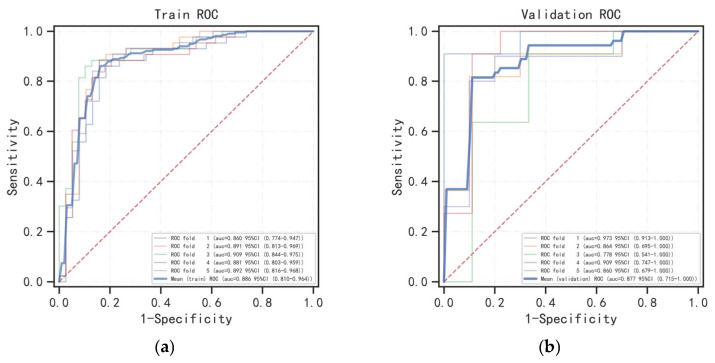

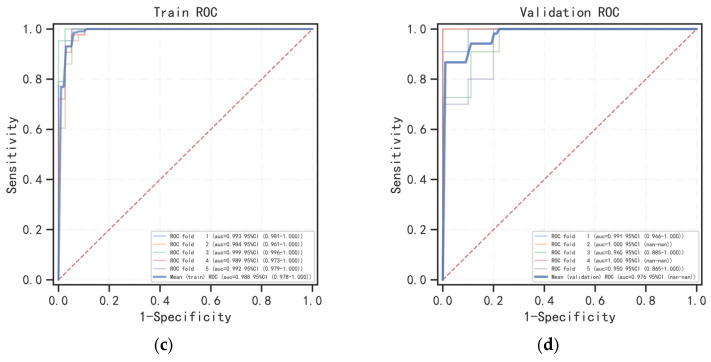
Five-fold and mean ROC curves based on the Radscore and Clinic–Radscore models in the training and validation groups. (**a**) ROC curves of the Radscore model in the training cohort. (**b**) ROC curves of the Radscore model in the validation cohort. (**c**) ROC curves of the Clinic–Radscore model in the training cohort. (**d**) ROC curves of the Clinic–Radscore model in the validation cohort.

**Figure 5:**
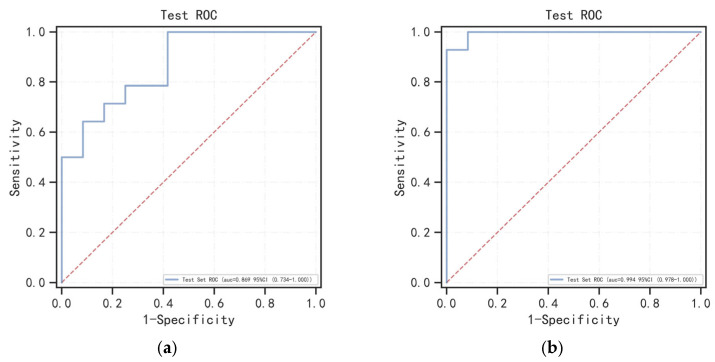

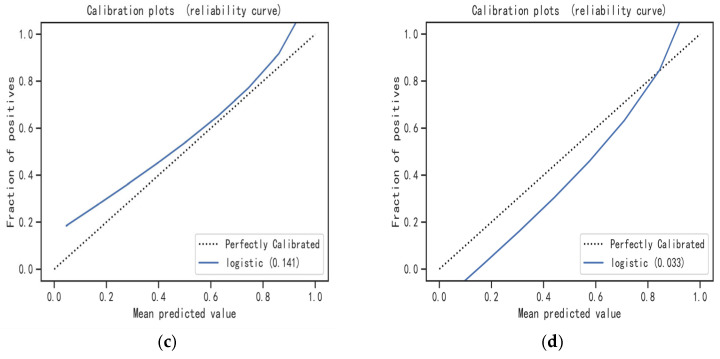
ROC curves and calibration curves based on the Radscore and Clinic–Radscore models in the test cohort. (**a**) ROC curves of the Radscore model in the test cohort. (**b**) ROC curves of the Clinic–Radscore model in the test cohort. (**c**) Calibration curves based on the Radscore model in the test cohort. (**d**) Calibration curves based on the Clinic–Radscore model in the test cohort.

**Figure 6:**
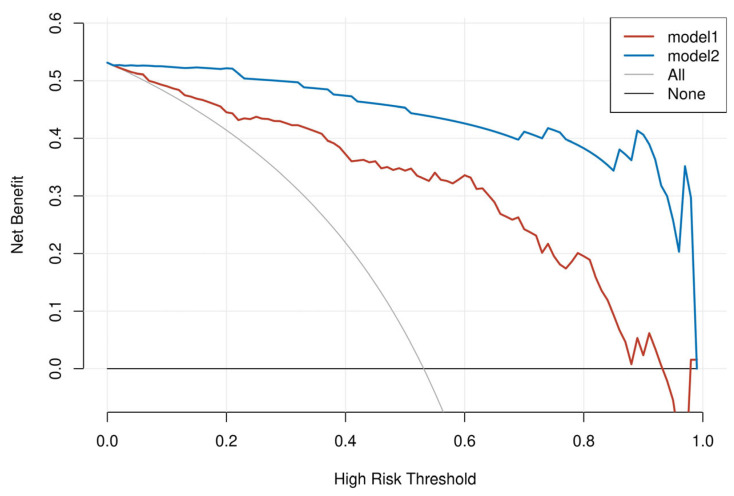
DCA of the Radscore and the Clinic–Radscore models in the test cohort. Model 1 is the Radscore model, and model 2 is the Clinic–Radscore model.

The 5-fold and mean ROC curves of the Radscore and Clinic–Radscore models in the training and validation cohorts are shown in Figure 4, and the 5-fold AUC values of the 5-fold ROC curves, as well as the 95% confidence interval (CI), are provided in Table 4. The mean AUC values of the mean ROC curves and the 95% CI, accuracy, sensitivity, specificity, and F1 score values are shown in Table 5. For the test cohort, the ROC curves of the Radscore ML model and Clinic–Radscore ML model are shown in Figure 5a,b. The AUC values of the ROC curves and the 95% CI, accuracy, sensitivity, specificity, F1 score, and the DeLong test values of the Radscore and Clinic–Radscore models are shown in Table 6. For the test cohort, the AUC value of the Radscore model was 0.869 [95% CI, 0.734–1.000], and the accuracy, sensitivity, specificity, and F1 score values were 0.731, 1.000, 0.583, and 0.900, respectively. The AUC value of the Clinic–Radscore model was 0.994 [95% CI, 0.978–1.000], and the accuracy, sensitivity, specificity, and F1 score values were 0.962, 0.929, 1.000, and 0.931, respectively. The DeLong test values for the comparison of the Radscore and Clinic–Radscore models in the test set was 1.859 (*p* = 0.063). The DeLong test revealed no significant difference in the diagnostic efficacy between the Radscore ML model and the Clinic–Radscore ML model, and the single Radscore also achieved good performance in the differential diagnosis between APAs and NAAs. The calibration curves of the Radscore and Clinic–Radscore ML model for the test cohort are shown in Figure 5c,d. The correction curve showed good agreement between the predicted and observed probabilities. The DCA of the Radscore ML model and the Clinic–Radscore ML model in the test set are shown in Figure 6. The DCA demonstrated that the Clinic–Radscore ML model had net benefits in distinguishing APAs from NAAs, outperforming the Radscore ML model.

**Figure 7:**
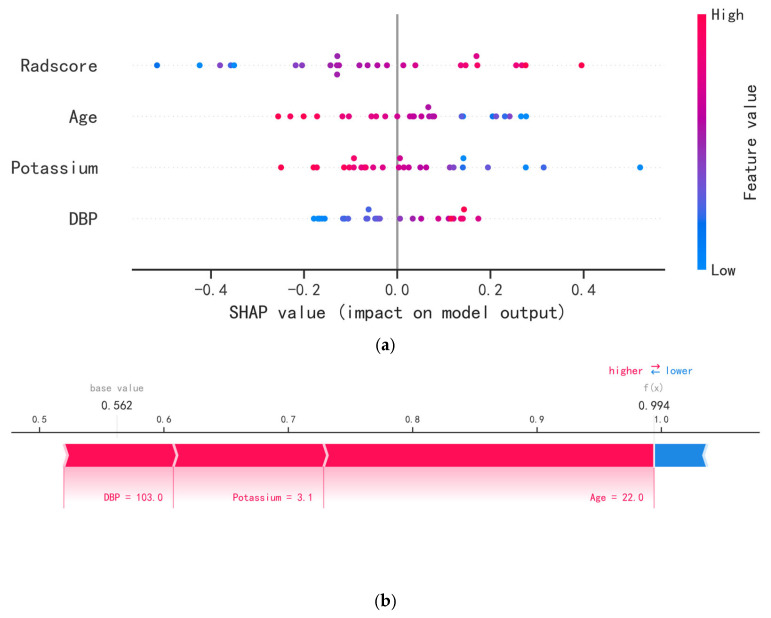
Summary plots for SHAP values. Each point represents a patient in each feature. (**a**) The SHAP summary of the degree by which the feature contributed to the risk of APA. (**b**) An example for predicting the risk of APA.

Figure 7a shows the SHAP summary plot of the Clinic–Radscore ML model. Four important features were arranged according to the contribution importance. The higher the SHAP value of a variable, the higher the risk of APA. The y-axis represents the order of the absolute values of the features’ coefficients, and the x-axis denotes the positive and negative directionality of the features’ coefficients. The blue to red colors denote the influence of a feature from low to high. The Radscore was the most important variable, whereas DBP was the least important. The Radscore and DBP had positive coefficients, whereas age and potassium had negative coefficients. This indicated that the higher the Radscore and DBP, the higher the risk of APA, and the lower the age and potassium level, the higher the risk of APA. Figure 7b shows an example for predicting the risk of APA. In this patient, the Clinic–Radscore logistic regression ML model predicted an APA risk of 0.994 (base value: 0.562). The probability of an APA was increased by DBP, potassium, and age. Finally, we generated an online model to facilitate clinical application evaluation. (https://www.xsmartanalysis.com/model/list/predict/model/html?mid=8107&symbol=416oCwZ9434368Jm1eE0, accessed on 18 September 2023).

## 4. Discussion

This study investigated the ability of a CECT-based radiomics model to distinguish APA from NAA. The predictive ability was accomplished by constructing a Clinic–Radscore model that combined radiomic features and baseline clinical variables. The CECT-based radiomics model exhibited a good ability to distinguish APA from NAA. The AUC value of the Radscore ML model in the test cohort (0.869) revealed a high degree of diagnostic efficacy. When the radiomics features were combined with clinical variables in the Clinic–Radscore ML model, the AUC in the test cohort further increased to 0.994. The specificity of the Radscore ML model was 0.583, and the specificity of the Clinic–Radscore ML model was 1.000. Because both NAA and APA are adenomas, it may be that the texture features of NAA and APA overlap. However, clinical characteristics such as hypokalemia and hypertension are specific for PA patients. The DeLong test showed no significant difference between the combined Clinic–Radscore model and the simple Radscore models in the test cohort. Collectively, these results indicate that compared with Clinic–Radscore ML model, the Radscore ML model alone has good predictive ability for the differential diagnosis of APA from NAA. However, the Clinic–Radscore ML model performed better than the Radscore ML model.

Among the baseline clinical variables, age, Time, SBP, DBP, potassium level, and Radscore significantly differed between the APA and NAA groups. The differences in age and potassium level were consistent with the findings reported by Chen and Bioletto et al., wherein the patients in the APA group were younger and had lower potassium concentrations [16,23]. However, in the current study, patients with normal baseline blood pressure were included because of the possibility of subclinical PA. Particularly, there was evidence of hypersecretion aldosterone in those patients with AI, but they did not develop clinical symptoms such as hypertension or hypokalemia. One review has described an intermediate phenotype of PA with normal blood pressure, and PA was diagnosed in 12% of AI patients with normal blood pressure and with normal serum potassium levels [17]. As many as 20% of those with refractory hypertension can develop APA, and the prevalence increases according to the severity of hypertension [24,25]. In the present study, the SBD and DBP levels of APA patients were higher than those of NAA patients. The Clinic–Radscore ML model also showed that the higher the level of DBP, the higher the risk of APA.

The aldosterone renin ratio and potassium level are independent predictors of PA [23]. In the current study, the Clinic–Radscore model was constructed based on a combination of clinical characteristics and radiomics features, the ability of radiomics to distinguish APA from NAA was confirmed, and an auxiliary diagnostic method was identified for the preliminary diagnosis and classification of AI. CT-based radiomics may reflect the amount of information that can be inferred from the lesion volume and lesion mask, as well as that of the microenvironment of the internal tissues. Radiomics features can be used to quantify the inner texture of lesions based on various metrics such as the gray-level co-occurrence matrix (GLCM), gray-level run length matrix, gray-level size zone matrix, and neighborhood gray tone difference matrix [26,27,28]. A previous study confirmed that a correlation exists between pathological features of the tumor and the GLCM [29]. In tumor cells, the positive immunostaining of the cytochrome P450 enzyme CYP11B2, also known as aldosterone synthase, can be used to distinguish APA from NAA [30]. However, the correlation between the radiomics and pathological features of APA and NAA require further investigation. One study reported that radiomics features were correlated with fat regulation and fat protein metabolism, biological oxidation, and gene expression [31]. The present study also found differences in certain radiomics features between those with APA and with NAA, including the following parameters: original_shape_Flatness, original_shape_SurfaceVolumeRatio. Those shape features denote the difference in APA and NAA in the aspect of shape. Some features were filtered through the wavelet, as follows: wavelet-LLH_firstorder_10Percentile, wavelet-LLH_gldm_LargeDependenceLowGrayLevelEmphasis, wavelet-LLH_glszm_ZoneEntropy, wavelet-LHL_glcm_Imc2, wavelet-LHL_ngtdm_Busyness, wavelet-LHH_glrlm_RunEntropy, wavelet-HHL_gldm_DependenceNonUniformityNormalized, wavelet-LLL_glcm_MaximumProbability. Some of these features represent the heterogeneity between textures; for example, ZoneEntropy indicates the uncertainty and randomness of an image [32].

Radiomics has been increasingly applied for diagnosing adrenal gland diseases in recent years; however, most studies have focused on its ability to identify benign, malignant, and low-fat tumors [13,33,34]. Relevant studies have been conducted on the function of preoperative adenomas in the field of pheochromocytoma [14,35,36]. However, only a few studies on the utility of radiomics in assessing the function of preoperative APA have been reported to date. A study by He et al. [15] used a radiomics model to predict the likelihood of unilateral adrenal nodules in patients with PA and APA, and good results were obtained for both the training and validation sets, with AUCs of 0.900 and 0.912, respectively. These results indicate that radiomics can predict the likelihood of a unilateral nodule being APA in patients with PA. This information is important because it can determine whether the adrenal tumor should be surgically removed during clinical treatment. For example, Akai et al. [37] used texture analysis to locate adrenal glands with increased aldosterone production to guide clinical treatment.

The findings of this study may be valuable in clinical practice. For patients with hypertension, adrenal imaging examinations are aimed at determining the lesion’s lateralization and whether it is benign or malignant. Unilateral adrenal tumors or malignant lesions can sometimes be cured through surgical interventions. However, traditional imaging information can only characterize benign and malignant adrenal tumors to a limited extent; in most cases, further targeted endocrine testing is needed to distinguish the endocrine function of adrenal tumors. This study confirms that radiomics-based approaches can determine the secretory function of AI tumors prior to surgery, which allows shunting in AI patients prior to further endocrine testing, reducing unnecessary endocrine testing, especially in patients with low risk of APA. The current study included patients with normal blood pressure with the aim of identifying potential subclinical PA according to radiomics analysis. Thus, we may detect potential APA in advance after CT examination, but before endocrine testing.

However, this study also had some limitations. First, only patients with CECT imaging data were included. Further studies are required to determine whether radiomics approaches based on unenhanced CT have a similar efficacy in distinguishing APA from NAA. And the manual segmentation of adrenal adenomas from CT images is complicated and prone to human error, which affects the accuracy of the model; automatic segmentation may improve this. Second, this study only compared patients with APA and with NAA, and did not compare patients with other types of potentially secretory AI, such as those with pheochromocytomas. Therefore, further studies are needed to evaluate adrenal tumors with potentially similar overlapping CT imaging features. Third, the sample size was small, and this was a single-center study; thus, the test cohort was from the same institution as the training cohort, and the patient population was relatively homogeneous. The model’s performance in generalizing different populations or settings is therefore unclear. This may need to be tested in different medical institutions and even in different populations. Fourth, this study was retrospective in nature and thus may have been prone to selection bias. Therefore, a prospective, large-scale, multicenter study is required to evaluate the validity of the model identified in this study. Fifth, in this study, we only discussed the performance of logistic regression ML model, and the performance of other classification models needs to be further investigated and compared.

## 5. Conclusions

The CECT-based radiomics ML model developed in this study exhibited high diagnostic efficacy in distinguishing APA from NAA based on radiomics features alone, and the diagnostic efficacy of the model further increased after incorporating baseline clinical characteristics. In the future, radiomics models may be constructed by artificial intelligence as a non-invasive, simple, and efficient technique to assist radiologists in the functional diagnosis of secretory AI. Models such as the one developed in this study can aid clinicians in the subtype diagnosis of cases with secretory functional adenoma and in follow-up evaluations of these patients with NAA. The Radscore model can help guide therapeutic decision making to achieve individualized precision medicine for patients.

## Figures and Tables

**Table 1:** Packages used in the development of machine learning models.

Package Classification	Package Name	Version	Usage
R	glmnet	4.1.2	LASSO
Python	sklearn	0.22.1	ROC
R	rmda	1.6	DCA
Python	shap	0.39.0	SHAP
Python	sklearn	0.22.1	Logistic regression ML

**Table 2:** Comparison of patient characteristics between the training and test cohorts.

Variables	Training (*n* = 102)	Test (*n* = 26)	χ^2^/t/z	*p* Value
Age, years	51.00 (40.75–57.00)	48.50 (36.50–54.25)	−1.301	0.193
Sex			0.927	0.336
Male	50 (49.02%)	10 (38.46%)		
Female	52 (50.98%)	16 (61.54%)		
Time ^1^	1.00 (0.00–6.25)	2.50 (0.13–8.50)	−1.456	0.145
SBP, mmHg	140.36 ± 20.05	139.15 ± 19.88	0.275	0.784
DBP, mmHg	88.00 (80.00–97.25)	83 (78.25–103.25)	−0.684	0.494
FPG, mmol/L	5.57 (4.93–6.97)	6.26 (5.44–7.91)	−1.963	0.050
Potassium, mmol/L	3.57 ± 0.68	3.51 ± 0.58	0.454	0.651
TC, mmol/L	4.04 (3.69–4.71)	4.54 (3.70–4.96)	−1.108	0.268
Scr, umol/L	68.50 (56.00–82.25)	63.50 (52.75–82.50)	−0.892	0.373
eGFR, ml/min/1.73 m^2^	100.35 (87.10–107.38)	102.10 (87.48–112.50)	−0.980	0.327
BMI, kg/m^2^	24.19 ± 3.51	24.27 ± 2.93	−0.104	0.917

^1^ Duration of time since the discovery of hypertension.

**Table 3:** Comparison of patient characteristics between the APA and NAA groups in the training cohort.

Variables	APA (*n* = 54)	NAA (*n* = 48)	χ^2^/t/z	*p*
Age, years	48.00 (36.75–54.00)	55.50 (51.00–57.75)	−3.965	<0.001
Sex			0.961	0.327
Male	24 (44.44%)	26 (54.17%)		
Female	30 (55.56%)	22 (45.83%)		
Time ^1^	3.00 (0.56–8.00)	0.00 (0.00–3.00)	−4.537	<0.001
SBP, mmHg	147 (134.00–165.50)	127.00 (121.25–142.75)	−3.692	<0.001
DBP, mmHg	94.56 ± 14.36	84.75 ± 10.39	3.980	<0.001
FPG, mmol/L	5.66 (4.93–7.08)	5.53 (4.92–6.48)	−0.674	0.050
Potassium, mmol/L	3.10 ± 0.54	4.10 ± 0.35	−11.243	<0.001
TC, mmol/L	3.91 (3.61–4.65)	4.19 (3.71–4.36)	−1.438	0.150
Scr, umol/L	68.50 (56.00–82.50)	68.50 (59.25–82.50)	−0.054	0.957
eGFR, ml/min/1.73 m^2^	101.75 (89.85–108.05)	99.50 (85.43–106.58)	−1.200	0.230
BMI, kg/m^2^	23.68 ± 3.43	24.77 ± 3.55	−1.585	0.116
Radscore	0.86 ± 0.76	−0.67 ± 1.02	8.455	<0.001

^1^ Duration of time since the discovery of hypertension.

**Table 4:** Five-fold AUC values of the five-fold ROC curves and the 95% CI of the Radscore and the Clinic–Radscore models in the training and validation cohorts.

Models	Groups	1-Fold AUC (95% CI)	2-Fold AUC (95% CI)	3-Fold AUC (95% CI)	4-Fold AUC (95% CI)	5-Fold AUC (95% CI)
Radscore	Training	0.860 (0.774–0.947)	0.891 (0.813–0.969)	0.909 (0.844–0.975)	0.881 (0.803–0.959)	0.892 (0.816–0.968)
	Validation	0.973 (0.913–1.000)	0.864 (0.695–1.000)	0.778 (0.541–1.000)	0.909 (0.747–1.000)	0.860 (0.679–1.000)
Clinic–Radscore	Training	0.993 (0.981–1.000)	0.984 (0.961–1.000)	0.999 (0.996–1.000)	0.989 (0.973–1.000)	0.992 (0.979–1.000)
	Validation	0.991 (0.966–1.000)	1.000 (nan–nan ^1^)	0.960 (0.885–1.000)	1.000 (nan–nan)	0.950 (0.865–1.000)

^1^ Denotes the AUC values achieved to 1.000.

**Table 5:** Mean AUC values of the mean ROC curves and the 95% CI, accuracy, sensitivity, specificity, and F1 score values of the Radscore and the Clinic–Radscore models in the training and validation cohorts.

Models	Groups	Mean AUC (95% CI)	Accuracy	Sensitivity	Specificity	F_1_
Radscore	Training	0.886 (0.810–0.964)	0.846	0.861	0.854	0.863
	Validation	0.877 (0.715–1.000)	0.823	0.869	0.871	0.854
Clinic–Radscore	Training	0.988 (0.978–1.000)	0.958	0.986	0.953	0.972
	Validation	0.976 (nan-nan ^1^)	0.921	0.964	0.938	0.946

^1^ Denotes the AUC values achieved to 1.000.

**Table 6:** Mean AUC values of the ROC curves and the 95% CI, accuracy, sensitivity, specificity, and F1 score values of the Radscore and the Clinic–Radscore models in the test cohort.

Models	AUC (95% CI)	Accuracy	Sensitivity	Specificity	F_1_	z	*p*
Radscore	0.869 (0.734–1.000)	0.731	1.000	0.583	0.900	1.859	0.063
Clinic–Radscore	0.994 (0.978–1.000)	0.962	0.929	1.000	0.931		

## Data Availability

The datasets used or analyzed during the current study and the online machine learning model are available from the corresponding author on reasonable request.
